# The Avahan Transition: Effects of Transition Readiness on Program Institutionalization and Sustained Outcomes

**DOI:** 10.1371/journal.pone.0158659

**Published:** 2016-07-19

**Authors:** Sachiko Ozawa, Suneeta Singh, Kriti Singh, Vibha Chhabra, Sara Bennett

**Affiliations:** 1 Department of International Health, Johns Hopkins Bloomberg School of Public Health, Baltimore, MD, United States of America; 2 Amaltas Consulting Private Limited, New Delhi, India; Simon Fraser University, CANADA

## Abstract

**Background:**

With declines in development assistance for health and growing interest in country ownership, donors are increasingly faced with the task of transitioning health programs to local actors towards a path to sustainability. Yet there is little available guidance on how to measure and evaluate the success of a transition and its subsequent effects. This study assesses the transition of the Avahan HIV/AIDS prevention program in India to investigate how preparations for transition affected continuation of program activities post-transition.

**Methods:**

Two rounds of two surveys were conducted and supplemented by data from government and Avahan Computerized Management Information Systems (CMIS). Exploratory factor analysis was used to develop two measures: 1) transition readiness pre-transition, and 2) institutionalization (i.e. integration of initial program systems into organizational procedures and behaviors) post-transition. A fixed effects model was built to examine changes in key program delivery outcomes over time. An ordinary least square regression was used to assess the relationship between transition readiness and sustainability of service outcomes both directly, and indirectly through institutionalization.

**Results:**

Transition readiness data revealed 3 factors (capacity, alignment and communication), on a 15-item scale with adequate internal consistency (alpha 0.73). Institutionalization was modeled as a unidimensional construct, and a 12-item scale demonstrated moderate internal consistency (alpha 0.60). Coverage of key populations and condom distribution were sustained compared to pre-transition levels (p<0.01). Transition readiness, but not institutionalization, predicted sustained outcomes post-transition. Transition readiness did not necessarily lead to institutionalization of key program elements one year after transition.

**Conclusion:**

Greater preparedness prior to transition is important to achieve better service delivery outcomes post-transition. This paper illustrates a methodology to measure transition readiness pre-transition to identify less ready organizations or program components in advance, improving the likelihood of service sustainability. Further research is needed around the conceptualization and development of measures of institutionalization and its effects on long-term program sustainability.

## Introduction

One of the greatest challenges of donor-led programs is to sustain activities and outcomes beyond the project funding period. Too often projects end abruptly when donor funding discontinues. This is particularly the case when the donor has supported service delivery through systems that are parallel to and separate from government’s own health systems. Sometimes donors invest in planning for a transition process whereby key elements of the program are handed over to local partners including government, but this kind of planned transition process is relatively rare [1]. Quantitative studies measuring the success of transitions are uncommon.

The challenges of transition are shared across many donor-led programs, and is a particularly pertinent question at this time given tempered growth in development assistance for health [2, 3] as well as for HIV/AIDS [4, 5], and plans to promote country ownership and transition within the U.S. President’s Emergency Plan for AIDS Relief (PEPFAR) [6–8]. This challenge is also relevant to ongoing graduation of countries from donor programs such as the Global Fund to Fight AIDS, Tuberculosis and Malaria [9] and Gavi, the Vaccine Alliance [10–12].

Transitions, if done well, provide an opportunity for donors to steward successful programs into the capable hands of local actors and integrate them into domestic country health systems. A successful transition involves formally handing over a program to one or more local partners and ensuring that the key outcomes of the program are sustained over time. An effective transition is often characterized by alignment and smooth transfer of key services, good communication about transition preparations and plans, and sufficient capacity among local program owners [1]. Preparations pre-transition are frequently described to be essential in this process [13, 14].

Transition may also involve a process of change, as donors and local actors may have different structures, management practices and organizational cultures. The extent of continuity may be captured by institutionalization, which considers the extent to which the initial program systems are integrated into organizational procedures and behaviors post-transition [15]. Institutionalization is seen to be important in program sustainability, considering the extent to which innovation and learning not only gets adopted and continued, but also gets reflected in institutional standards and norms that govern multiple organizations within the broader health system [15]. To date, there has been a dearth of empirical work to measure and assess the degrees of institutionalization post-transition [16].

Increasing attention has been given in recent years to examine whether various outcomes of health programs are sustained [17, 18]. A review of empirical literature identified 125 studies on sustainability which examined the performance of health programs after initial implementation [19]. Many studies reported that partial sustainability was more common than continuation of the entire program or intervention. However, the wide array of studied outcomes and durations made it difficult for reviewers to generalize the overall extent to which new programs and practices are sustained. The review also found the majority of studies to be retrospective, with few studies employing rigorous methods of evaluation. The large majority of studies took place in high income countries.

This study uses data from an assessment of the transition of the Avahan India HIV/AIDS prevention initiative to investigate how activities undertaken to prepare Avahan for transition affected continuation of program activities post-transition. Prior to transition, Avahan was fully supported by the Bill and Melinda Gates Foundation, whose staff managed grants or contracts with a range of international and national Non-Government Organizations (NGOs) [20]. These organizations in turn contracted with smaller NGOs and Community Based Organizations (CBO) to provide HIV/AIDS prevention services for key populations known as Targeted Interventions (TIs) [21, 22]. Key populations in this study included female sex workers and/or high-risk men having sex with men. Transition experiences from the community perspective were examined separately through qualitative studies [23].

Service delivery of Avahan was transitioned to the Government of India in three increasingly larger rounds of transition in 2009, 2011 and 2012. This study examines the transitions that took place in tandem in southern Indian states. Post-transition, the government took on responsibilities to fund and manage TIs, where they must adhere to national guidelines set by the National AIDS Control Organization (NACO), while being accountable to State AIDS Control Societies (SACS). TIs were transitioned to four different SACS where application of NACO norms varied by state in practice, making them a heterogeneous group. While the majority of TIs transitioned in original forms, some TIs had split because they were too large for NACO guidelines, or separated by key populations. Other TIs merged because they were too small for NACO norms, or discontinued. Other papers provide a fuller overview of the Avahan transition process, and the strengths and weaknesses of this process [1, 24–27].

This study examines the transition by focusing on three core elements: 1) how well prepared programs were prior to transition, 2) whether key elements of the program were institutionalized post-transition, and 3) whether outcomes were sustained through the transition. In particular, we examined relationships between transition readiness, institutionalization and outcomes to provide insights in the effectiveness of transition planning. We hypothesized that transition preparedness may explain what happens post-transition in terms of (i) institutionalization of key program elements one year after transition and (ii) sustained program delivery. This paper is unique in developing measures of transition preparedness and institutionalization, using data from both before and after transition to analyze the relationship between these variables, and examining linkages with sustainability of service outcomes.

## Materials and Methods

Surveys were carried out in five southern states of India where Avahan was transitioned to local partners: Andhra Pradesh (now Andhra Pradesh and Telangana), Karnataka, Maharashtra, and Tamil Nadu. We conducted two rounds each of the transition readiness and institutionalization surveys. The transition readiness survey was conducted at the time of transition, whereas the institutionalization survey was carried out 12 to 18 months post-transition. All of the TIs from the 2011 round of transition were surveyed, whereas we used a list of transitioning TIs stratified by state to randomly select TIs from the larger 2012 round of transition. Data from 2011 and 2012 rounds of transition were combined together in the analysis to achieve sufficient sample size.

The transition readiness survey examined a range of indicators that sought to assess how well prepared the TIs were for transition. Through a literature review and conceptual framework of a transition logic model which was developed to evaluate the Avahan transition [1], we identified three elements of transition readiness: 1) capacity, 2) alignment, and 3) communication. Capacity indicators captured key operations of the TI, such as linkages made with government health facilities, formation of key population community groups and functioning of crisis response committees [28]. Alignment indicators measured levels of preparations made by the TI towards meeting NACO norms in areas such as team structure, budgeting and reporting. Communication captured whether staff were informed about the transition, transition plans incorporated staff inputs and project coordinators received training for the transition. Indicators were considered important where there were government norms to assess the extent to which TIs are aligned, as well as questions to understand how well prepared the TIs were for the transition in terms of capacity and communication [24, 29]. In addition to interview questions, the survey also included a review of data in the Avahan Computerized Management Information System (CMIS) from which we abstracted relevant indicators. For each indicator gathered from interviews and documents, we defined 3 levels (0 = low, 1 = medium and 2 = high) of transition readiness based on how well prepared TIs were for transition. For example, we classified the extent to which TIs met NACO norms, established linkages with government health services or had informed staff about the transition. The transition readiness survey captured 21 indicators, including 18 which were asked to TI managers and 3 which were abstracted from CMIS data.

The institutionalization survey examined whether transitioned TIs had institutionalized characteristics of the original program post-transition. Specifically, we asked whether transitioned TIs were continuing to regularly practice certain characteristics of the Avahan program. Core Avahan characteristics were identified from an earlier Delphi study, of which 13 of the 17 features were considered to be relevant post-transition [30]. The institutionalization survey included 18 interview questions which asked how frequently TIs carry out identified Avahan practices (i.e. regularly, sometimes, never). We then asked about Avahan practices in 20 statements using a five-point Likert scale, including whether the transition experience went smoothly overall. Additional 20 questions asked whether these practices changed due to the transition, which institution (i.e. government, TI or others) brought about the changes and whether these changes were for the better, worse, or made no difference. Interview questions were supplemented by a review of the government’s CMIS for relevant indicators.

Program outcome data were gathered from our surveys, the Avahan CMIS database and TI proposal documents. To observe changes pre- and post-transition, we selected indicators which were recorded in both the Avahan CMIS (for the pre-transition period) and the government’s CMIS (for the post-transition period). Available outcome indicators were limited based on the data that TIs gathered and reported. As a primary outcome indicator, we used the average percentage of key populations contacted by peer educators per month. Specifically, Avahan CMIS and government CMIS had collected the number of female sex workers and high-risk men having sex with men who were newly or repeatedly contacted by peer educators using counseling materials. These contacts were divided by the number of key populations served by each TI. As a secondary outcome indicator, we examined the average number of condoms distributed per key population per month. Sizes of key populations for each TI were obtained from TI proposal documents or the Avahan CMIS database [31]. Pre-transition data were abstracted from the Avahan CMIS database for 18 TIs to supplement the government CMIS data received from TIs. Panel analysis was used to plot observations. Heteroskedasticity was assessed using the Breusch-Pagan / Cook-Weisberg test [32, 33]. For each outcome a fixed effects model was developed with robust standard errors.

Measures of transition readiness and institutionalization were separately constructed through exploratory factor analysis. Based on the literature, we hypothesized the transition readiness measure to be multi-dimensional capturing elements of capacity, alignment and communication, and developed 3 sub-scales for this measure. However, there was no prior literature to inform the items to construct the scales. The literature also did not provide evidence to suggest the dimensionality of the institutionalization measure. Items for both scales were factor analyzed using iterated principal factor extraction with Varimax orthogonal rotation. The number of factors was determined using a scree test with eigenvalue greater than one. Items were analyzed and retained based on factor loadings, item-to-total correlation and inclusion of diverse elements of the concepts. Construct validity was examined by correlation analyses against other theoretically related constructs. Internal consistency was assessed by Cronbach’s alpha.

**Fig 1:**
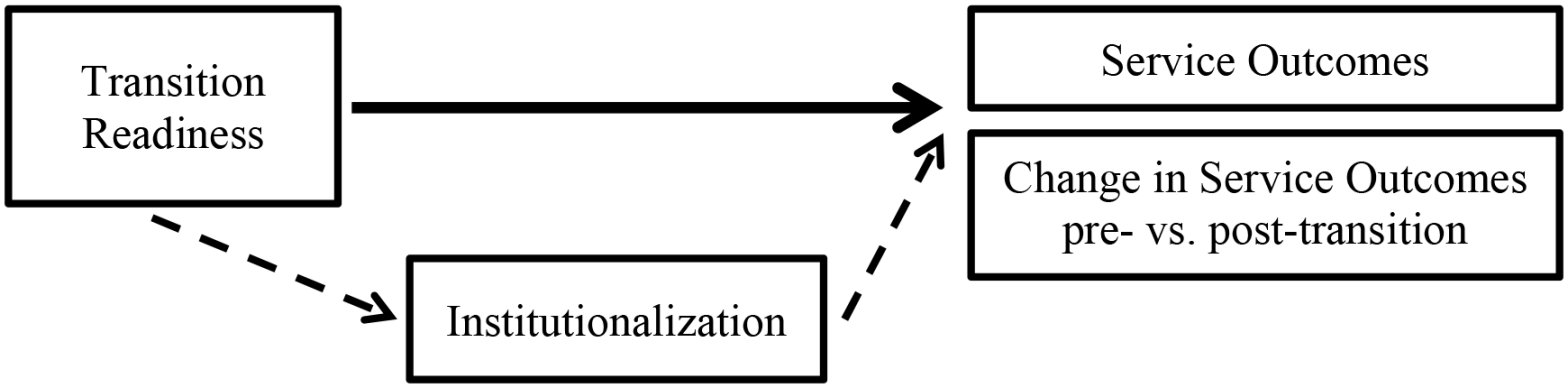
Relationship between transition readiness, institutionalization and service outcomes.

Using the two developed measures, ordinary least squares regression was subsequently carried out to examine the relationship between transition readiness and program outcomes directly, and indirectly through institutionalization (Fig 1). Relationships between transition readiness subscales and outcomes were also separately examined. For both outcome indicators, we examined the relationship of average outcomes 12 months pre-transition and 6 months post-transition. Univariate analyses were followed by multivariate analyses controlling for rounds of transition, whether the TI had split at the time of transition, NGO or CBO status of the TI, target key population (female sex workers, high-risk men having sex with men, or both), and state.

This study was ethically reviewed by Johns Hopkins School of Public Health Institutional Review Board (IRB No. 3157) and exempted as the study collected data from key informants but not data about individuals. In India, it was reviewed and approved by YRG Care Institutional Review Board (IRB No. 1423). Participants provided written informed consent to participate in the study.

## Results

### Transition readiness

Transition readiness data were collected from 80 TIs including all 27 TIs that transitioned in 2011 (100%) and 53 of the 155 TIs that transitioned in 2012 (34%). Our sample was representative of different states (34% Andhra Pradesh/Telangana, 29% Karnataka, 24% Maharashtra and 14% Tamil Nadu), type of organizations (64% NGO, 36% CBO) and TI support for key populations (84% served female sex workers, 57% served men having sex with men) (Table 1).

**Table 1:** Characteristics of Surveyed Targeted Interventions.

	Transition Readiness (N = 80)		Institutionalization (N = 70)*	
	No. of TIs	(%)	No. of TIs	(%)
**State**				
Andra Pradesh /Telangana†	27	34%	24	34%
Maharashtra	19	24%	21	30%
Karnataka	23	29%	17	24%
Tamil Nadu	11	14%	8	11%
**Organization Type**				
NGO	51	64%	49	70%
CBO	29	36%	21	30%
**Year of Transition**				
2011	27	34%	28	40%
2012	53	66%	42	60%
**Key Population Served**				
FSW	34	43%	31	44%
MSM	13	16%	12	17%
Composite	33	41%	27	39%
**Split TI**				
Split	29	36%	21	30%
Not Split	51	64%	49	70%

* Sample size for the institutionalization survey was smaller due to TIs that discontinued, split up, merged or delayed the time of transition.

^**†**^ The state of Andhra Pradesh split into Andhra Pradesh and Telangana after the data collection was completed (in June 2014).

CBO: Community-based organizations; FSW: female sex workers; MSM: men having sex with men; NGO: Non-government organizations.

Overall, many TIs scored high across transition readiness indicators, demonstrating various adjustments made to meet NACO norms, build capacity and communicate prior to the handover. Most TIs had appropriately communicated and aligned the reporting systems (average score of 1.88 out of 2), staff structure (1.95 out of 2) and budgets (1.88 out of 2) prior to transition. Where transition readiness scores were lower, we found that some TIs delayed alignment on procurement of medicines due to buffer stocks (0.96 out of 2), or had removed condom outlets in hotspots because of vandalism and privacy concerns (1.21 out of 2). Scores were also lower for visits to Integrated Counselling and Testing Centers (ICTC) post-referral (1.10 out of 2), where government CMIS records suggested that 80% of referrals were actually seen at ICTC centers compared to 100% requested by NACO (Table 2).

**Table 2:** Original Transition Readiness Indicators.

Transition Readiness Indicators		Low (0)*	Medium (1)	High (2)	Mean†	SD	Item-to-Total Correlation
1	Does the NGO/CBO have a linkage with Government ICTC services?	No linkage established	Most cases are referred to government services	Most cases referred receive these services	1.61	0.58	0.86
2	Does the NGO/CBO have a linkage with ART centers?	No linkage established	Most cases are referred to government services	Most cases referred receive these services	1.64	0.48	0.82
3	Does the NGO/CBO have a linkage with TB screening centers?	No linkage established	Most cases are referred to government services	Most cases referred receive these services	1.64	0.53	0.81
4	Has there been any change in the reporting format, and are you sending any reports to SACS/District AIDS Control Societies?	No change in reporting format	SACS formats discussed but not all introduced	Following all SACS formats	1.88	0.46	0.54
5	Do all identified hotspots have condom outlets?	None have condom outlets	Some hotspots have condom outlets	All hotspots have condom outlets	1.21	0.67	0.50
6	Have the staff been informed about the transition?	Not informed	Has been discussed	Transition plans have been developed with staff inputs	1.71	0.46	0.46
7	Has there been any change in the budget as per NACO/SACS guidelines?	No change, still following Avahan budget	Some changes were made to the budget	Following NACO/SACS budget guidelines	1.88	0.37	0.44
8	What percentage of key populations who are referred actually visit the ICTC?	Poor coverage (<50%)	Over 50% of key populations referred actually visit the ICTC (<100%)	100% of key populations referred actually visit the ICTC	1.10	0.85	0.36
9	What is the present ratio of outreach worker to key population?	Ratio was not previously measured	Ratio is measured and approaching that of SACS	Following the SACS ratio	1.89	0.36	0.33
10	Have project coordinators/directors received training on TI guidelines for transition as recommended by SACS?	No training received	Training has been planned, but has not yet taken place	Training has been received	1.61	0.75	0.29
11	Has there been any change in the TI team structure, and are you following the SACS/NACO guidelines?	No change in team structure	Some changes were introduced	Following SACS TI structure	1.95	0.22	0.24
12	What is the coverage of identified key populations with regular contact (two contacts each month)?	Some key populations contacted in last month (<30%)	Over 30% contacted in last month (<60%)	60% or more contacted in the last month	1.93	0.31	0.24
13	Have community members at the hotspots formed crisis response committees?	No committees formed	Committees formed, but less than 30% of members meet every month	Committees formed, and 30% or more members meet every month	1.81	0.45	0.24
14	Has there been any change in the condom procurement process?	No change in condom procurement process	Some changes are made in the condom procurement processes	All condom procurement is done through channels suggested by SACS	1.53	0.80	0.24
15	Does the NGO/CBO procure STI syndromic management medicines as per NACO/SACS guidelines?	Avahan supply chain is still in place	Some changes are made in the procurement processes as suggested by NACO/SACS	STI syndromic management medicines are procured as per NACO/SACS guidelines	0.96	0.96	0.23
*16*	*What is the coverage of syndromic management for key populations with STI*?‡	*Poor coverage (<50%)*	*Over 50% of key populations with STI syndromes receive treatment (<100%)*	*100% of key populations with STI syndromes receive treatment*	*1*.*43*	*0*.*78*	*0*.*19*
*17*	*Is the NGO/CBO following the STI syndromic management guideline of NACO*?‡	*Avahan guidelines are still in place*	*Some changes are made according to NACO guidelines*	*Following STI syndromic management guidelines of NACO*	*1*.*79*	*0*.*54*	*0*.*17*
*18*	*What is the present ratio of peer educators to key populations*?‡	*Ratio was not previously measured*	*Ratio is measured and approaching that of SACS*	*Following the SACS ratio*	*1*.*94*	*0*.*24*	*0*.*10*
*19*	*Has the NGO/CBO been able to form groups at the community level*?‡	*Groups have not been formed*	*Group formation in process*	*Groups have been formed*	*1*.*89*	*0*.*39*	*0*.*10*
*20*	*Have project coordinators/directors received training on program management for transition as recommended by SACS*?‡	*No training received*	*Training has been planned*, *but has not yet taken place*	*Training has been received*	*1*.*55*	*0*.*79*	*0*.*03*
*21*	*Has there been any change in the Avahan method of micro-planning*?‡	*Avahan method of micro-planning is still in place*	*Some changes are made to micro-planning*	*Micro-plan follows NACO guidelines and is updated every quarter*	*1*.*49*	*0*.*81*	*-0*.*02*

* Responses were categorized as low (0), medium (1) or high (2) levels of transition readiness based on NACO norms.

^**†**^ Mean, standard deviation and item-to-total correlations are based on the full sample (n = 80) across two rounds.

^‡^ In developing the transition readiness scale, 6 items with low item-to-total correlation (below 0.20; rows 16–21 in *Italic*) were removed.

CBO: Community-based organization; ICTC: Integrated counselling and testing center; NACO: National AIDS Control Organization; NGO: Non-governmental organization; SACS: State AIDS Control Societies; SD: Standard deviation; STI: Sexually Transmitted Infections.

Based on an item analysis of 21 transition readiness items, those with negative factor loadings and item-to-total correlations below 0.2 were removed. This resulted in 15 items in the transition readiness scale explaining 81% of the variance. Items were summed unweighted, with a mean score of 24.34 (standard deviation 3.09) and a range of 16–29. Indexed to a 0–100 scale, the mean was 81.11 (standard deviation 10.30). The scale demonstrated acceptable internal consistency with a Cronbach’s alpha of 0.73. Some evidence toward construct validity was found where the transition readiness scale was correlated with TIs reporting smooth transition experience at r = 0.29 (p = 0.03).

Exploratory factor analysis revealed 3 factors which appeared to reflect the three elements of transition readiness from the literature: capacity (eigenvalue 3.92), alignment (eigenvalue 2.57), and communication (eigenvalue 2.08), although not all items were aligned with the factor with which we had originally associated them. Three subscales were then developed for sub-analyses corresponding to the 3 identified factors. Factor 1 (capacity) was associated with 4 items, factor 2 (alignment) with 4 items, and factor 3 (communication) with 7 items (Table 3).

**Table 3:** Transition Readiness Scale Items and Factor Loadings.

		Factor I Capacity*	Factor II Alignment	Factor III Communication
1	Does the NGO/CBO have a linkage with Government ICTC services?	**0.96**†	0.14	0.05
2	Does the NGO/CBO have a linkage with ART centers?	**0.95**	0.02	0.04
3	Does the NGO/CBO have a linkage with TB screening centers?	**0.94**	0.08	-0.05
4	Has there been any change in the reporting format, and are you sending any reports to SACS/District AIDS Control Societies?	0.39	**0.50**	0.05
5	Do all identified hotspots have condom outlets?	-0.50	0.05	**0.08**
6	Have the staff been informed about the transition?	0.27	0.13	**0.39**
7	Has there been any change in the budget as per NACO/SACS guidelines?	0.18	**0.80**	-0.02
8	What percentage of key populations who are referred actually visit the ICTC?	-0.15	-0.23	**0.02**
9	What is the present ratio of outreach worker to key population?	0.11	**0.29**	0.29
10	Have project coordinators/directors received training on TI guidelines for transition as recommended by SACS?	0.16	0.05	**0.32**
11	Has there been any change in the TI team structure, and are you following the SACS/NACO guidelines?	0.19	-0.13	**0.35**
12	What is the coverage of identified key population with regular contact (two contacts each month)?	**0.10**	0.09	0.03
13	Have community members at the hotspots formed crisis response committees?	-0.01	**0.42**	0.18
14	Has there been any change in the condom procurement process?	0.03	0.11	**0.66**
15	Does the NGO/CBO procure STI syndromic management medicines as per NACO/SACS guidelines?	-0.22	-0.23	**0.54**

* Results are based on an iterated principal factor analysis with 3 factors using orthogonal varimax rotation, based on 15 items with item-to-total correlation above 0.2, for the entire sample (n = 80).

^**†**^ Values are bolded to indicate which factor the item is associated with.

### Institutionalization

Institutionalization data were collected from 70 TIs, including 28 TIs that transitioned in 2011 and 42 TIs that transitioned on time in 2012. Although we sought to conduct the transition readiness assessment and the institutionalization survey in the same sample of TIs, several TIs discontinued (1 TI in 2011 round), split up (2 TIs in 2011 round), merged (1 TI in 2012 round) or delayed the time of transition (10 TIs in 2012 round), resulting in differences in sample sizes. However, distributions of sampled TI characteristics were largely not affected (Table 1).

Frequency of practice of Avahan characteristics were examined on a scale from 0 to 2 (0 = never, 1 = sometimes or 2 = regularly). Most TIs reported actively using data for program planning (average score of 1.99 out of 2), regularly using the pictorial micro-planning tool (1.96 out of 2), and practicing rigorous performance monitoring of outreach workers (1.99 out of 2) [see Table 4]. However, some Avahan characteristics were less regularly maintained post-transition, such as providing flexibility on budgets (0.41 out of 2) and allowing exceptions to operating norms (0.89 out of 2). Another Avahan characteristic to have on-time, adequate and uninterrupted flow of funds to the grassroots level was often not continued, where 7 TIs (10%) reported regularly and 31 TIs (44%) sometimes facing challenges with cash flow that affected their operations during the 12 months post-transition.

**Table 4:** Original Institutionalization Indicators.

	Institutionalization Indicators	Avahan Characteristic	Mean*	SD	Item-to-Total Correlation	Factor Loadings‡
1	Does a committee of community members oversee the program?	Committees of community members that oversee the program	1.70	0.57	0.50	0.25
2	During the past year has the NGO/CBO received supervisory visits from DAPCU or SACS or TSU?	Extensive onsite supportive supervision provided by managers and technical area specialists	1.9	0.3	0.48	0.56
3	Do you find supervisory visits to be a good opportunity for you to discuss solutions to any problems you may face?	Extensive onsite supportive supervision provided by managers and technical area specialists	1.87	0.38	0.47	0.71
4	During the past year, have you ever had any problem with cash flows from the SACS that has affected your operations?†	On-time, adequate and uninterrupted flow of funds and commodities to the grassroots level	1.36	0.66	0.46	0.39
5	Does SACS allow any exceptions to operating norms (other than budget) such as the PE/ORW ratio, based on realities on the ground?	Flexible management style that facilitates response to local needs	0.89	0.73	0.45	0.27
6	Do you find that the crisis response system works?	Community-led crisis response management	1.84	0.40	0.45	0.18
7	Does your NGO/CBO actively use data to monitor progress in the program?	Active use of data at all levels for planning and regular review of program delivery	1.99	0.12	0.44	0.67
8	During the past year has your TI always had sufficient stock of commodities, such as condoms or medicines?	On-time, adequate and uninterrupted flow of funds and commodities to the grassroots level	1.67	0.5	0.40	0.06
9	Do peer educators and outreach workers receive skills and leadership training (beyond general orientation training)?	Need based systematic training to enhance peer outreach workers' skills and leadership	1.44	0.65	0.32	0.06
10	Do peer outreach workers use pictorial micro-planning to facilitate their mapping of most at risk populations?	Pictorial micro-planning tool for peer outreach workers	1.96	0.2	0.28	0.09
11	Has SACS provided any flexibility on budget, based on realities on the ground?	Flexible management style that facilitates response to local needs	0.41	0.6	0.26	0.19
12	Do you find that SACS/NACO advocates on behalf of key population programs?	Support to service delivery through strong advocacy programs at national and state level	1.01	0.88	0.25	0.05
*13*	*Is the performance of peer outreach workers monitored rigorously*?§	*Rigorous performance monitoring of peer outreach workers by staff supervisors and community committee*	*1*.*99*	*0*.*12*	*0*.*23*	*-*
*14*	*Do you plan for saturated coverage of small pockets of key populations*?§	*Saturation coverage of even smaller pockets of key populations*	*1*.*93*	*0*.*26*	*0*.*22*	*-*
*15*	*Are the training needs of peer educators and outreach workers assessed*?§	*Need based systematic training to enhance peer outreach workers' skills and leadership*	*1*.*90*	*0*.*39*	*0*.*20*	*-*
*16*	*Have you supported community groups and organizations*?§	*Strong focus on fostering community groups and organizations*	*1*.*76*	*0*.*46*	*0*.*19*	*-*

* Mean, standard deviation and item-to-total correlations are based on the full institutionalization sample (n = 70) across two rounds. All responses are categorized regularly (2), sometimes (1) and never (0).

^**†**^ Negative coding was applied (never having cash flow problems were given a score of 2).

^‡^ Results are based on an iterated principal factor analysis with one factor, based on 12 items.

^§^ In developing the institutionalization scale, 4 items with low item-to-total correlation (below 0.25; rows 13–16 in *Italic*) were removed.

Factor analysis revealed one dominant factor with an eigenvalue of 2.35. Among the 16 original items, 4 were removed due to negative factor loadings and low item-to-total correlation below 0.25 (Table 4). Dropped items included those with high average values with little variation, where items were less useful to differentiate TIs in a scale. The institutionalization scale was then developed using 12 items, with an average score of 18.04 and standard deviation of 2.59. On a scale from 0–100, this corresponds to a mean of 75.17 and standard deviation of 10.79. The scale explained 52% of the variance and demonstrated moderate internal consistency with a Cronbach’s alpha of 0.60. In assessing construct validity, the institutionalization scale was appropriately but weakly negatively correlated with TIs reporting that the overall program has changed significantly as compared to pre-transition r = -0.17 (p = 0.15).

### Sustained Outcomes

TIs with outcomes data both pre- and post-transition were limited even after combining multiple data sources (government CMIS data obtained from TIs, Avahan CMIS database and TI proposal documents). Data were available for 55 TIs (79% of the institutionalization sample) for the primary outcome indicator: average percentage of key populations contacted by peer educators per month. Data were obtained for 64 TIs (91% of institutionalization sample) for the secondary outcome indicator: average number of condoms distributed per key populations per month.

Both outcomes were relatively stable between 12 months pre-transition and 6 months post-transition. While there was a minor drop in the average percentage of key populations contacted by peer educators one month post-transition, there were no trends observed comparing pre- and post-transition in the levels of either indicator based on the fixed effects model (Table 5). For both outcomes, the data was found to be heteroskedastic, where variance of the data reduced post-transition (p<0.001).

**Table 5:** Sustainability of outcomes 12 months pre-transition and 6 months post-transition.

		Fixed Effects Model				
Outcomes	Indicators	Coeff.	Robust SE	*p-*value	*95% Conf*. *Interval*	
Key Population Coverage	Change in average percentage of key populations contacted by peer educators per month	0.65	0.37	0.08	-0.08	1.38
	Average percentage of key populations contacted by peer educators per month	73.68	3.66	<0.01*	66.34	81.01
Condom distribution	Change in average number of condoms distributed per key population per month	0.05	0.32	0.88	-0.59	0.68
	Average number of condoms distributed per key population per month	38.13	3.18	<0.01*	31.78	44.49

* This is a p-value of a constant in a regression, testing if the mean is significantly different from zero.

### Effect of transition readiness and institutionalization on sustained outcomes

Based on a regression analysis, we observed a statistically significant relationship between transition readiness and sustained outcomes post-transition, for both key population coverage (2.47, p<0.01) and condom distribution (2.17, p = 0.03). The transition readiness measure was also predictive of outcomes comparing 6 months pre- and post-transition, for key population coverage (0.03, p<0.01) and condom distribution (0.04, p = 0.02). Examining the subcomponents of the transition readiness measure, this relationship was strongest across the dimensions of alignment and communication (Table 6). None of the variables we controlled for were statistically significant, including rounds of transition, NGO or CBO status of the TI, target key population or state.

**Table 6:** Relationships between transition readiness, institutionalization and outcomes.

	Transition Readiness												Institutionalization		
	Overall			Capacity			Alignment			Communication			Overall		
Dependent Variables*†	Coeff.	SE	*p-*value§	Coeff.	SE	*p-*value	Coeff.	SE	*p-*value	Coeff.	SE	*p-*value	Coeff.	SE	*p-*value
**Key Population Coverage**‡	**2.47**	**0.83**	**< 0.01**	3.48	1.93	0.08	**6.87**	**2.30**	**< 0.01**	1.69	1.23	0.18	3.06	1.69	0.08
**Condom Distribution**	**2.17**	**0.98**	**0.03**	-0.50	2.06	0.81	3.97	2.84	0.17	**3.27**	**1.31**	**0.02**	0.25	1.33	0.85
**Key Population Coverage pre- vs. post-transition**	**0.03**	**0.01**	**<0.01**	0.03	0.02	0.12	**0.05**	**0.02**	**0.04**	**0.03**	**0.01**	**0.02**	-0.002	0.02	0.91
**Condom Distribution pre- vs. post-transition**	**0.04**	**0.02**	**0.02**	0.04	0.03	0.26	**0.12**	**0.04**	**0.01**	0.03	0.02	0.23	0.007	0.02	0.75
**Institutionalization**	0.01	0.09	0.91	0.16	0.19	0.40	-0.08	0.26	0.76	-0.03	0.13	0.79			

* Based on a univariate ordinary least squares regression where rows show dependent variables and columns illustrate independent variables.

^†^ Analysis controlled for rounds of transition, NGO or CBO status of the TI, target key population, and state.

^‡^ Analysis is based on key population coverage (n = 55), condom distribution (n = 64) and institutionalization (n = 70).

^§^ Relationships where p-value <0.05 are highlighted in bold.

However, this relationship between transition readiness and service delivery outcomes did not hold through institutionalization. Specifically, the transition readiness measure was weakly associated with institutionalization (0.01, p = 0.91) and significant associations were not observed between institutionalization and service outcomes (3.06, p = 0.08 for key population coverage; 0.25, p = 0.85 for condom distribution). While transition readiness was predictive of sustained outcomes, there were no indirect effects observed through institutionalization.

## Discussion

We find that transition readiness can explain sustained program delivery in an HIV/AIDS prevention program in India handed over from a donor to the local government. Specifically, TIs with greater preparedness prior to the transition were more likely to achieve better service delivery outcomes post-transition, and this was also true for dimensions of the transition readiness scale, including alignment and communication. Although the four distinct SACS implemented NACO norms with some variation, we found that the relationship between transition readiness and sustained outcomes did not significantly differ by state.

Yet we also found that transition readiness did not necessarily lead to institutionalization of key program elements one year after transition, nor was institutionalization predictive of sustained program delivery. These results may be explained partly by difficulties in measuring the construct of institutionalization. While we developed our institutionalization measure based on the core characteristics of the Avahan program identified through a Delphi study [30], the measure may not have sufficiently captured the construct of institutionalization as indicated by the scale’s moderate internal consistency and modest proportion of explained variance. In addition, our study design did not capture the extent of Avahan practices prior to transition, thus making it difficult to assess whether there was truly a decline in the prevalence of these practices.

Furthermore, there appears to be some conceptual misalignment between measures of alignment with government norms in the transition readiness scale vis-à-vis the continuation of specific Avahan program characteristics post-transition captured through institutionalization. For example, ensuring on-time, adequate and uninterrupted flow of commodities was viewed to be an Avahan characteristic, yet TIs had to change their procurement with government supply systems to be transition ready, which likely led to interruptions in commodities supplied. Conversely, TIs that were better prepared for transition, as embodied by well-informed and skilled staff and high institutional capacity, may have been more inclined to innovate and change as compared to TIs with less capacity. Indeed, there appeared to have been substantial change in the TIs surveyed since transition, and much of this change was positively perceived by TI managers. As programs evolve over time, the linkages between transition readiness, institutionalization and sustained impact become more complex and would benefit from dynamic systems modeling [34].

While there was some evidence to suggest that the three elements of transition readiness—capacity, alignment and communication—based on a literature review and conceptual framework [1] was supported by the data, we found it to be far from conclusive. For instance, the loadings of the three factors did not align entirely with our initial conception of the three sub-scales. In addition, raising the item-to-total correlation cutoff level in the analysis removed variables and collapsed the subscales into one factor. This provides good fodder for future research to look into the sub-components of the transition readiness measure.

Our main findings support the largely grey literature in this area describing transitions and sustainability [18, 19, 35–37]. It also adds to the limited measurement literature around transitions and institutionalization [38–41]. While Goodman et al provides an eight-factor model on institutionalization of health promotion programs, we found it difficult to apply this in our study in the context of an evolving and transitioning project with a short follow-up time period [40]. The mostly descriptive accounts of how communication and transition planning affect the overall success of transition of HIV/AIDS programs [13, 14, 42] are supported through our analysis.

Some additional study limitations are important to note. First, the study was limited by the small sample size of TIs that took part in the program transition. While we surveyed 100% of TIs that transitioned in round 1 and 34% from round 2, the sample size of 80 TIs limit the power in our statistical analyses. Moreover, some TIs were split or discontinued, making it difficult to track their status post-transition. The quality of administrative data gathered through CMIS was also a limiting factor, where some data points were missing and restricted possible service delivery outcomes for analysis. Where possible, we supplemented the government CMIS data by extracting data from other sources such as the Avahan CMIS database and TI protocol documents. Finally, our analysis examined institutionalization one-year post transition, which may not have provided sufficient time for program characteristics to be reflected in institutional standards and norms, or capture the types of feed-back loops in the diffusion of service innovations [16]. Despite these limitations, this paper adds value by evaluating the transition of a large-scale donor-led program to local counterparts both prior to and after the transition.

This study contributes to the literature by developing approaches to measure transition readiness and institutionalization in the context of a health program transitioning from a donor to local partners. While our measure particularly of institutionalization appears imperfect, this study suggests that it may be practical and useful to assess transition readiness quantitatively prior to transition. While such measures should be tailored to fit the nature of the program being transitioned, it appears that they may be a good indicator of service sustainability post-transition, as well as helping to identify needs for further transition preparation.

## Conclusion

This study offers important lessons for future transitions of donor programs. Specifically, we illustrate a methodology to measure transition readiness prior to transition, which could be predictive of service delivery outcomes post-transition. Such a measure could identify less ready organizations or program components in advance, improving the likelihood of service sustainability. Further research is needed around the conceptualization and development of measures of institutionalization and its effects on long-term program sustainability.
